# Polycyclic aromatic hydrocarbons in *Mullus surmuletus* from the Catania Gulf (Sicily, Italy): distribution and potential health risks

**DOI:** 10.1007/s11356-020-11052-z

**Published:** 2020-10-09

**Authors:** Rosaria Ornella Bua, Annalinda Contino, Alessandro Giuffrida

**Affiliations:** Dipartimento Di Scienze Chimiche, Università Degli Studi di Catania, Viale Andrea Doria 6, 95125 Catania, Italy

**Keywords:** PAHs, GC-MS, *Mullus surmuletus*, Risk assessment, Sicily

## Abstract

Different specimens of *Mullus surmuletus* from the Catania Gulf (Sicily) were sampled and analysed for the quantification of 16 priority polycyclic aromatic hydrocarbons (PAHs) in order to evaluate the distribution of these pollutants and the potential human health risks associated to the consumption of fish. The determined PAHs concentration values ranged from 0.25 to 6.10 ng/g wet weight and the most abundant PAHs detected were lower molecular weight (LMW) compounds with 2 to 3 rings. Relying on the PAHs concentration values, on the consumption data and on the total toxic equivalent (TEQ), the incremental lifetime cancer risk (ILCR) was assessed and its calculated value (2.97 × 10^−7^) is far below the “maximum acceptable risk level” (ARL), suggesting a low potential carcinogenic risk on consuming *M. surmuletus* for local population. Even though the study shows a quite low contamination level in *M. surmuletus*, intensive monitoring programmes are still highly needed in order to provide a better picture of the PAHs distribution in Catania Gulf and of the human health risk linked to fish consumption.

**Electronic supplementary material:**

The online version of this article (10.1007/s11356-020-11052-z) contains supplementary material, which is available to authorized users.

## Introduction

Polycyclic aromatic hydrocarbons (PAHs) are widely distributed environmental contaminants either from anthropogenic and natural sources whose origin can be petrogenic (Pampanin 2017) or pyrogenic (Balmer et al. 2019). Most of the combustion by-products present in smoke are PAHs (Khalili et al. 1995; Gustafson et al. 2008; Bignal et al. 2008), a class of compounds with toxic, mutagenic and carcinogenic characteristics (Baird et al. 2005; Xue and Warshawsky 2005). Among PAHs, the IARC (International Agency for Research on Cancer) has classified benzo(a)pyrene as carcinogenic to humans (Group 1); dibenzo(a,h)anthracene as a probable human carcinogenic (Group 2A); and naphthalene, benzo(a)anthracene, chrysene, benzo(b)fluoranthene, benzo(k)fluoranthene and indeno[1,2,3-cd]pyrene as possible human carcinogenics (Group 2B) (IARC 2010, 2014; Idowu et al. 2019; Savchenko et al. 2019). Several other PAHs, including acenaphthene, fluoranthene, phenanthrene and pyrene, were instead listed in Group 3-Unclassifiable as carcinogenic in humans.

In the marine environment, PAHs are usually adsorbed on small solid particles suspended, as well as on sediments, and thus they may enter the tissues of fish and other marine organisms through the food chain. However, whereas until 2007 the scientific community thought that the biomagnification and bioaccumulation processes, in edible marine organisms, can introduce PAHs in humans through the diet (Domingo et al. 2007), it has recently been demonstrated (Baali and Yahyaoui 2019; Franco and Lavado 2019) that PAHs are quickly metabolised in fresh fish and do not accumulate in the muscle meat. In fact, due to the inducible enzymes of the cytochrome P450 family (Frasco and Guilhermino 2002), in particular CYP1A, that catalyse the addition of an oxygen atom to the PAH molecules, these last become highly polar conjugated metabolites that are then excreted into the urine or the bile for rapid elimination over the gastrointestinal tract. This rapid metabolism in fish make concentrations of parent PAHs insignificant in muscle and other tissues resulting in the modification of Regulation (EC) No 1881/2006 (EC Regulation 2006) in Regulation (EU) No 835/2011 (EC Regulation 2011), which states that a maximum level for PAHs in fresh fish is no longer appropriate. In fact, PAHs are generally detected in trace quantities in contaminated marine organisms and, therefore, the previous limit of 2 μg kg^−1^ for benzo(a)pyrene in fresh fish has been repealed, maintaining only the limits for smoked products. The distribution of PAHs in marine organism strongly depends on the lipid content of the organisms themselves, feedings habitat and trophic level. In fact, it has been reported that the distribution of PAHs compounds differs from species to species (Rose et al. 2012). However, the accumulation of PAHs and its metabolite in fish can negatively affect the health of fish (Logan 2007; Balk et al. 2011) as well as the health of human populations who consume these fishes. Thus, in the last few years, the human health risks resulting from dietary intake of PAHs have been assessed using different equations that properly combine different factors (Zhao et al. 2014; Barhoumi et al. 2016; Oliva et al. 2017; Moslen et al. 2019; Soltani et al. 2019; Yu et al. 2019; Apiratikul et al. 2020). Many of these risk assessments are based on the benzo(a)pyrene equivalent (BaPeq) concept, e.g. the toxicity of PAHs in food is calculated from the concentration values of benzo(a)pyrene and their toxicity equivalency factors (TEF). Using the BaPeq, age, body weight and carcinogenic potency of benzo(a)pyrene, the incremental lifetime cancer risk (ILCR) (also referred to as the excess cancer risk) associated to ingestion of food contaminated with PAHs can be estimated (Xia et al. 2010; Bandowe et al. 2014; Oliva et al. 2017; Wang et al. 2019b; Fang et al. 2020).

In this context, the area investigated in this paper, the Catania Gulf, is of particular interest, being characterized either by anthropogenic pressures, including industrial and municipal impacts and high maritime traffic (Astiaso Garcia et al. 2013; Viola et al. 2017), as well as by natural pressures, such as the Etna volcanic activity (Conte et al. 2015, 2016). In fact, volcanic ashes, in contact with seawater, release macronutrients and traces of metals that become bioavailable for marine phytoplankton, which “responds” to the supply of nutrients in a couple of days and up to a few weeks with a more intense flowering (Jones and Gislason 2008; González-Vega et al. 2020). In particular, the essential nutrients for the Mediterranean marine ecosystem (such as nitrogen, phosphorus, silica, iron and zinc) can influence the primary productivity of the sea up to a distance of 700 km from Etna. If we consider that the Mediterranean Sea is generally a sea poor in nutrients, this contribution of macronutrients due to the presence of the volcano, even at a considerable distance, renders the sea of Catania Gulf extremely full of fish, in such a way that this food is not only largely consumed by the local population but, at the same time, the largest amount of fish found at the local market comes from the Gulf itself. Thus, the volcano has a positive impact on the fishing area, but, on the other hand, could increase the concentration of PAHs in the environment and in fish itself. Even though the extremely interest of the area, only few scientific papers have been published on different species of fish caught in Catania Gulf (Conte et al. 2016; Ferrante et al. 2018). In the present study, the fish *Mullus surmuletus* has been selected to monitor PAHs pollution in Catania Gulf, since this species is widely distributed in the Mediterranean Sea. Furthermore, it has great ecological importance and has been suggested as bioindicator for assessing the contamination status and ecological risks by the European monitoring program (EU 2010), in addition to being highly consumed from the local population. The determination of the PAHs concentration values was carried out on properly selected tissues, and by using the QuEChERS method that is still widely used for the determination of PAHs in marine organisms (Duedahl-Olesen et al. 2020; Man et al. 2020). To the best of our knowledge, this is the first study that evaluate the levels of PAHs in *M. surmuletus* collected from Catania Gulf and that assess possible human health risks arising from the consumption of PAH contaminated fishes, allowing to actually shed light on the “true” health risk for the population of this unique area.

## Materials and methods

### Sampling

Specimens of *M. surmuletus* (*n* = 40) were sampled at the local fish market in Catania. The sampling took place in April 2019 before the spawning season. All information about the habitat and the characteristics of this species were collected from the online database www.fishbase.org. Consulting the growth curve of Von Bertalanffy (age-size relation) (Mukadder Arslan 2013), fishes of 3 years were sampled (length cm, 18.5 ± 1.8) and biometric data (length and weight) were recorded for every fish (Table 1). Dissection was performed after arriving in the laboratory with a stainless-steel knife, the internal organs were removed and muscle tissues with skin were isolated from the mid-body dorsal area. After that, samples were homogenized using a mixer and pooled, each pooled sample being made up of five specimens and a total of eight composite samples were stored at − 20 °C until the extraction process for PAH analysis.

**Table 1:** Biometric data, habitat, feeding mode and number of pooled individuals during the sample processing of the investigated fish species collected from the Catania Gulf, Sicily, Italy

Fish species	Red Mullet – *Mullus surmuletus*
Habitat	Demersal
Sedentary	Medium
Trophic level	3.5
Bathymetric distribution	5–409 m
Medium lipid content	5%
Length (cm)	18.5 ± 1.8
Weight (g)	75 ± 13.8
Total number of samples	40
Total pooled samples analysed	8

### Chemicals and reagents

Acetonitrile (purity > 99.9%), isooctane (purity > 99.0%) and anhydrous calcium chloride (CaCl_2_) were obtained from Sigma-Aldrich.

Supel™ QuEChERs products were prepackaged in anhydrous packages for EN 15662 containing 4 g magnesium sulfate (MgSO_4_), 1 g sodium chloride (NaCl), 1 g sodium citrate, and 0.5 g disodium citrate sesquihydrate and 5 g sodium bicarbonate (NaHCO_3_). dSPE tube, containing 900 mg MgSO_4_, 150 mg primary secondary amine (PSA) and 150 mg C18, were purchased from Sigma-Aldrich.

A 200 µg/mL standard stock solution of 16 PAHs includes naphthalene (NA), acenaphthylene (ACY), acenaphthene (AC), fluorene (FL), phenanthrene (PHE), anthracene (AN), fluoranthene (FLA), pyrene (PY), benzo(a)anthracene (BaA), chrysene (CH), benzo(b)fluoranthene (BbF), benzo(k)fluoranthene (BkF), benzo(a)pyrene (BaP), diBenzo(a,h)anthracene (DBahA), benzo(g,h,i)perylene (BghiP), indeno (1,2,3-cd)pyrene (IP) were obtained from Absolute Standards, Inc. As internal standards (IS), the naphthalene-d8, acenaphthene-d10, phenanthrene-d10, chrysene-d12, perylene-d12 standard mix (Absolute Standards, Inc.) was used, each component at 2000 μg/mL in methylene chloride.

A 1 µg/mL working solution of all 16 PAHs was prepared in isooctane. Calibration mixtures in the concentration ranges of 5, 10, 25, 50 and 100 ng/mL and with 25 ng/mL of internal standards were prepared by successive dilutions of the working solution in hexane.

A standard mixture of PAHs at 1 µg/mL in acetonitrile was used as a spiking solution for 10 ng/g spiking level in the recovery experiments.

### Sample extraction

The pooled muscle samples of *M. surmuletus* fishes were extracted by slightly modifying the extraction and the clean-up procedures reported by Wong et al. (2010). Briefly, 5 g of homogenized fillet were spiked with perdeuterated PAHs surrogate standard solution described in the “Materials and methods” section, added to 10 mL of acetonitrile and mixed by vortex. Then, 4 g of anhydrous MgSO_4_, 1 g NaCl, 5 g NaHCO_3_ and 0.5 g C_6_H_6_Na_2_O_7_ × 1.5 H_2_O (Supel™ QuEChERs) were added and the resulting mixture was mixed again. The extract was then centrifuged for 5 min at 5000 rpm and, subsequently, 5 mL of the acetonitrile layer was transferred into a tube and freezed at − 20 °C for 3 h to remove fatty acids. The extract was centrifuged for 5 min at 5000 rpm, and the supernatant was transferred into a tube containing 1 g of CaCl_2_ for the removal of free fatty acids co-extracted. The last clean-up step was carried out using dSPE (900 mg MgSO_4_, 150 mg PSA and 150 mg C18). The 15 ml dSPE tubes were vigorously vortex for 30 s and centrifuged for 5 min at 5000 rpm.

Finally, the supernatant was evaporated under nitrogen, and the residue was redissolved in 250 μl of isooctane.

### GC-MS analysis

The analyses of the extracted samples were performed using a GC coupled with a single quadrupole mass spectrometer (Thermo Finnigan Trace GC Ultra DSQ) managed by the Xcalibur software (ver 1.4 SR1). The analytical separations were performed using an Agilent J&W DB-5MS, 30 m, 25 mm ID, 0.25 μm capillary column. The carrier gas (helium) flow was set at a constant rate of 1 mL/min; and the injector, the ion source, and the MS transfer line temperatures were set at 280 °C. The injected volumes were 1 μL in splitless mode. The GC oven temperature program was set at initial temperature of 70 °C (1-min hold), ramped to 300 °C at 6 °C/min (5-min hold), resulting in a total run time of 44 min.

The selected ion monitoring (SIM) parameters and the average retention time for all the compounds tested that are specified in Supplementary Table S1.

### Quality assurance and quality control

In order to ensure quality control, blank samples, subjected to the same procedure of extraction previously described, but without adding fish tissue, were regularly analysed. The linearity and recovery were checked using homogenized muscle tissues of codfish, previously analysed as blank matrix.

To check the linearity, five calibration solutions were used to spike the codfish homogenates before the extraction procedure. Concentration ranges were 10–100 μg/L (10, 25, 50, 75, 100 μg/L) for PAHs and each level was determined in duplicate. Calibration curves were prepared by plotting the peak area ratio of the compound signal to the IS versus nominal concentration. The internal standard assigned for each compound was reported in Table S2. A good linearity was obtained, with values of the correlation coefficient *R* above 0.99.

Recovery was determined within the same analytical session by preparing 3 sets of samples. In the first set named “spiked,” blank codfish samples were spiked with the PAHs at 10 μg/kg and their labelled IS before the extraction step; in the second set named “post-spiked,” working solutions of the analytes were added (at the same concentration) on the blank codfish extracts; the third set, i.e. the “blank matrix” was the codfish sample extracted without any spiking. The recovery of the analytes was calculated by the ratio between the analyte concentration determined after its extraction (spiked) and that determined on the spiked extract (post-spiked) and the concerning data are reported in Table S2. The reproducibility and recovery were < 15% as CV% and between 80 and 120%, respectively.

The limits of quantification (LOQ) were estimated as 10σ (IUPAC criterion) and was about 0.25 ng/g for each congener.

The recovery data were used to quantify the concentrations of PAHs in the samples of *M. surmuletus* by using an external standard calibration curve. Quality controls (CQ) were performed by monitoring the recovery of spiked codfish within the same analytical sessions. Concentrations were calculated as ng/g (ppb) wet weight (ww).

### Data analysis

Basic descriptive statistical analyses were carried out using Microsoft Excel (vs. 1908) and the distribution of the total PAHs levels (∑PAHs, the sum of the 16 individual PAHs analysed) was checked for normality using the Shapiro–Wilk test. Analytes with a detection frequency below 30% in all the examined samples were excluded from statistics (e.g. sums and relative abundances), whereas for analytes with a higher detection frequency, the values below the method quantification limits (LOQ) were set to half their respective LOQ.

PAHs were grouped into three different classes according to the number of the rings: low molecular weight PAHs (LMW-PAHs) including 2–3 ring PAHs (NA, ACY, AC, FL, PHE, AN,), medium molecular weight PAHs (MMW-PAH) including 4 ring PAHs (FLA, PY, BaA, CH), and high molecular weight PAHs (HMW-PAH) including 5–6 ring PAHs (BbF, BkF, BaP, DBahA, BghiP, IP).

Furthermore, considering the IARC classification, the sum (∑cPAHs) of the carcinogenic (Group 1), probable (Group 2A) and possible (Group 2B) carcinogenic PAHs, was evaluated.

### Human health risk assessment

According to US-EPA (2000a), the toxicity equivalent factor (TEF) was used to calculate the total toxic equivalent (TEQ) in order to convert individual PAH component to an equivalent concentration of BaP, as follows:

$$ \mathrm{TEQ}=\sum \mathrm{TEFi}\times Ci $$

where *C*_*i*_ is the concentration of the individual PAH in fish muscle (ng/g ww), and TEFi is the corresponding toxic equivalency factor related to BaP (Nisbet and LaGoy 1992).

The daily dietary intake (DDI) due to fish consumption was evaluated according to the following equation:

$$ \mathrm{DDI}=C\times \mathrm{IR} $$

where *C* is the concentration of PAHs (∑_16_PAHs) and IR is the ingestion rate of fish (19.25 g/day), as the mean ingestion rate for the Italian population specific for demersal fish species supplied by FAOSTAT “Food Supply - Livestock and Fish Primary Equivalent” database online.

The incremental lifetime cancer risk (ILCR) associated with lifetime dietary exposure to the PAHs was calculated using the following equation (Bandowe et al. 2014; Wang et al. 2019b):

$$ \mathrm{ILCR}=\frac{\mathrm{TEQ}\times \mathrm{IR}\times \mathrm{ED}\times \mathrm{EF}\times \mathrm{SF}\times \mathrm{CF}}{\mathrm{BW}\times \mathrm{AT}} $$

where TEQ represents the BaP-equivalent concentration, IR is the ingestion rate (g/day), ED is the exposure duration (year), EF the exposure frequency (day/year), SF is the cancer oral slope factor of BaP, CF is the conversion factor of mg to ng, BW is the body weight (kg) and AT is the averaging time (day).

The values used in the ILCR calculation were set as follows: BW = 70 kg; IR = 19.25 g/day; AT = 25,550 day (70 year× 365 day/year); ED = 30 year for adults; EF = 365 day/year (US EPA 1989); SF = 7.3 (mg/kg/day)^−1^ for BaP; CF = 10^−6^.

## Results and discussion

### PAHs levels and distribution

The concentration values of PAHs found in the *M. surmuletus* analysed samples, as well as the total PAHs content (∑_16_PAHs), are reported in Table 2. The average concentration values ranged from 0.25 to 6.10 μg/kg ww for each congener, whereas the concentration value of ∑_16_PAHs was 19.49 μg/kg.

**Table 2:**
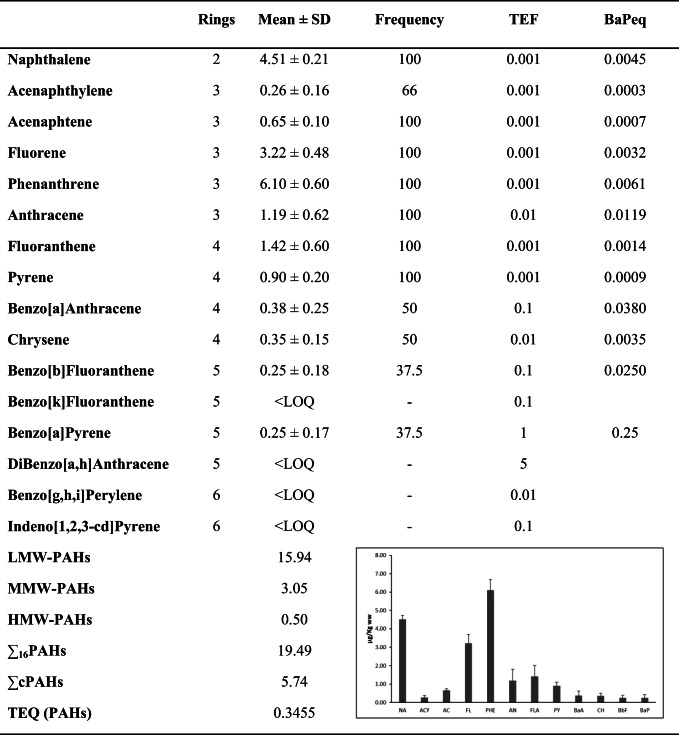
Average concentration values (μg/Kg ww) of PAHs, frequency of the presence of individual PAH (%) in the pooled samples, TEF values adopted from Nisbet and LaGoy (1992) and toxicity equivalent average values of BaP (BaPeq) (μg/Kg) in pooled fish samples of *M. surmuletus* from Catania Gulf, Sicily. In the inset a graphical representation of the data is reported where the error bars correspond to the standard deviation SD

Benzo[k]fluoranthene, diBenzo[a,h]anthracene, benzo[g,h,i]perylene and Indeno[1,2,3-cd]pyrene were never detected, whereas seven of the 16 target PAHs were found in all muscle pool samples (Table 2). Interestingly, benzo(a)anthracene and chrysene were detected in over 30% of the samples, while benzo(b)fluoranthene and benzo(a)pyrene, this last recognized as the most carcinogenic PAHs based on IARC classification, were found in only 3 of the 8 pools analysed and always at concentrations near the LOQ (0.25 μg/kg).

Since several factors contribute to the levels of pollutants in fish, such as uptake from the water column, diet, sediments, metabolism, partitioning and elimination, the comparison among the data obtained by different studies carried out at different sites is not straightforward. However, several studies were carried out in Sicily: Oliveri et al. (2012) analysed the muscle tissues of *Mullus barbatus* sampled in Lampedusa, Saija et al. (2016) investigated the accumulation of POPs in *Thunnus thynnus* from the Straits of Messina, Di Bella et al. (2018) detected persistent organic pollutants in farmed Sicilian sea bass and Ferrante et al. (2018) detected PAHs in three species (*Sardina pilchardus*, *Solea solea* and *Donax trunculus*) caught in the Catania Gulf. In particular, in the paper of Oliveri et al., the muscle tissues of a species similar to *M. surmuletus* was analysed, and it is thus possible to properly compare the two sets of data. Interestingly, the mean PAHs concentrations found in this study (Table 2) are lower than those found in Lampedusa (∑PAHs 26.47 μg kg^−1^) (Oliveri et al. 2012). Furthermore, these authors found higher levels of naphthalene (3.52 ± 6.13 μg kg^−1^ w.w.) and acenaphthylene (13.16 ± 3.44 μg kg^−1^ w.w.), whereas in this work, acenaphthylene was detected in traces and the most abundant PAHs are phenanthrene, naphthalene and fluorene. This different PAHs distribution probably relies on the different anthropogenic input to which marine environment is subjected. In fact, the intense maritime traffic is the primary PAHs source of contamination of the Sicilian Channel area, that originate, not only from the partial combustion of the fossil fuels, the combustion of biomass, the municipal waste, the accidental spills and the decomposition of the organic matter, but also from the vessel traffic (Oliveri et al. 2012).

**Fig. 1:**
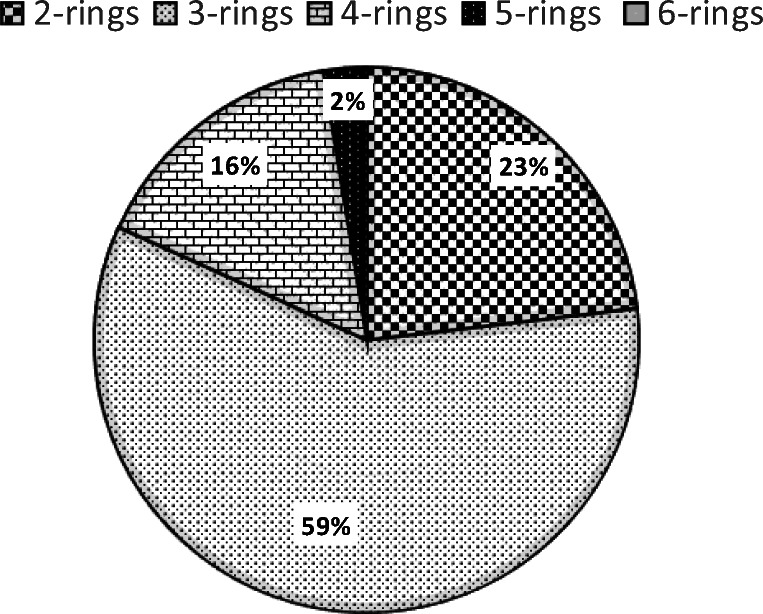
Percentage distribution (%) of PAHs based on the number of rings in pooled muscle samples of *Mullus surmuletus* from the Catania Gulf, Sicily

The composition profile (%) based on the number of rings of PAHs identified in the pooled fishes is reported in Fig. 1. The most abundant detected compounds are the low molecular weight PAHs (LMW-PAHs), accounting for the 81.8% of total PAHs with percentages of 23.1% and 58.6% for the two rings and for the three rings compounds, respectively. Instead, MMW-PAHs and HMW-PAHs only accounted for 15.7% and 2.5% of total PAHs, respectively. Among the detected PAHs, phenanthrene was the most abundant congener reflecting the distribution patterns of PAHs in seawater reported in some studies conducted in Mediterranean Sea (Berrojalbiz et al. 2011; Vecchiato et al. 2018). Phenanthrene, which is one of the main compounds of crude oil, is listed in Group 3-Unclassifiable as carcinogenic in humans by IARC (IARC 2010). The second most abundant congener detected was naphthalene, which is classified as possible carcinogen to humans (Group 2B) (IARC 2010).

These results are in agreement with those of several studies that report that PAHs found in living organisms are generally those containing two or three rings (Pointet and Milliet 2000; Binelli and Provini 2004; Mashroofeh et al. 2015; Dsikowitzky et al. 2016; Ranjbar Jafarabadi et al. 2019; Wang et al. 2019a; Fang et al. 2020). In fact, the accumulation of PAHs in fish is strongly influenced by several factors, including the PAHs solubility and bioavailability, molecular weight, and exposure route. Two to 3 rings PAHs show a greater solubility in water and a relatively slow metabolism, suggesting that the abundance of these compounds in the fish muscles is likely due to their uptake through the gills (Bautista et al. 2019). Noteworthy, several studies have shown a negative correlation between the accumulation of PAHs in several species and their log *K*_ow_, which lead to a higher bioavailability of LMW-PAHs (Dsikowitzky et al. 2016; Oliva et al. 2017; Honda and Suzuki 2020). On the other hand, HMW-PAHs bind to the sediments and to organic colloidal particles. Thus, they show lower concentration values in water that make them less bioavailable for their uptake through the gills, even though they could be absorbed from the sediments and from the organic particles present in the water columns via the digestive tract. Furthermore, in fish HMW-PAHs have a higher metabolism rate than the lower molecular weight ones (e.g. up to 99% of HMW-PAHs can be converted to metabolites within 24 h of uptake) and thus no accumulation of HMW-PAHs is usually observed (Varanasi et al. 1989) in these organisms. In fact, even though Frenna et al. (2013) reported high concentration values of 4 to 5-rings PAHs in Mediterranean Sea sediments sampled in different sites of Sicily, in the present study these compounds accounted for only 15.7% and 2.5% of the total PAHs detected in the investigated benthic fishes from the Catania Gulf, Sicily. This trend is also in agreement with the results reported in a study conducted on samples pooled in the Adriatic Sea, which reports the proportion of LMW-PAHs, MMW-PAHs, and HMW-PAHs residuals in fish to be of 62%, 37% and 1%, respectively (Perugini et al. 2007). This percentage composition seems to indicate a petrogenic origin. However, due to the higher metabolism speed and the consequent biotransformation of HMW-PAHs in fish, the pattern and distribution of PAHs themselves could be altered in fish tissues. Therefore, the accumulation of LMW-PAHs in fish muscle could be an artefact indeed due to the faster metabolic rate of HMW-PAHs (Varanasi et al. 1989; Oliva et al. 2017) and thus further investigations are highly needed.

### Human health risks assessment

The main route of human exposure to marine PAHs is through the marine living organisms that enter the human diet. Considering the IARC classification, the sum (∑c PAHs) of the carcinogenic (Group 1), probable (Group 2A) and possible (Group 2B) carcinogenic PAHs, was evaluated and it accounts for 29.5% of total PAHs. NA, classified as possible human carcinogenic (Group 2B), was the most abundant compound, while BaP (carcinogenic to human, Group 1), was detected only in three of the analysed fish muscle samples.

The toxicity equivalent of BaP (TEQ BaP) value was 0.3455 ng/g ww, which is lower than the screening values for the BaPeq (0.677 ng/g ww) suggested by USEPA for human fish consumption (US-EPA 2000b).

The Human Health Risk linked to fish consumption was evaluated using the concept of the daily dietary intake (DDI) and results to be 375.19 ng/day ww, which is slightly lower than those reported by Ferrante et al. (2018) for other demersal fishes collected in the same study area, the Catania Gulf. Analogously, the calculated ILCR index value is quite low (2.97 × 10^−7^), considering that an ILCR of 1 × 10^−5^ is regarded as the “maximum acceptable risk level” (ARL) (US-EPA 2000b).

Thus, the preliminary results reported in this paper suggest a potential low carcinogenic risk linked to the consumption of *M. surmuletus* for local population. However, the assessment of carcinogenic risks posed by fish consumption requires deeper investigation and should be carried out by taking into account the multiple pathways of the biotransformation of PAHs (Johnson-Restrepo et al. 2008) and the potential presence of other pollutants (Pompa et al. 2003; Wei et al. 2011).

## Conclusion

In this study, the detection and the determination of the concentration values of 16 PAHs in muscle fillet of *M. surmuletus*, caught in the Catania Gulf, was carried out. Seven of the 16 target PAHs were found in all pooled muscle samples of *M. surmuletus*, with phenanthrene, naphthalene and fluorene being the most abundant 2 to 3 rings PAHs. Interestingly, benzo(a)pyrene, which is recognized as the most carcinogenic PAH, based on the IARC classification, was found in only 3 of the 8 pooled samples analysed and always at concentrations near the LOQ. The concentration values of PAHs in muscle fillet from *M. surmuletus* were lower than those detected in other studies conducted on similar species living in the Mediterranean Sea.

In agreement with the data reported in the literature, the analysis of the composition profile, based on the number of rings, shows a greater occurrence of LMW-PAHs than HMW-PAHs. Considering that the distribution of PAHs in fish could be altered by the higher metabolism speed and consequent biotransformation of HMW-PAHs, further investigations are highly needed for the identification of pollution sources.

As regards the health risk, the carcinogenic PAHs accounted for 29.5% of total PAHs detected and naphthalene, which belongs to the IARC Group 2B (possible carcinogenic to humans), is the most abundant. Thus, the data shown in the present study, even though preliminary, contribute to obtain a more comprehensive picture of the distribution of PAHs in the Catania Gulf and provides as well an overview of the potential health risk due to the human consumption of *M. surmuletus* in this unique area. The calculation of the ILCR index value that falls well below the “maximum acceptable risk level,” suggests low potential human health risks through consumption of *M. surmuletus*.

Since the concentration values of LMW-PAHs are not negligible, continuous monitoring biota programmes, including also species having similar characteristics as bioindicators, should be carried out in order to shed light on the PAHs distribution and to evaluate the risk for human health associated to fish consumption in the Sicilian region. In this context, this study, which is the first report on the distribution of PAHs in *M. surmuletus* caught in the Catania Gulf, could represent a baseline for the monitoring of this area.

## Electronic supplementary material

Table S1 (DOCX 14 kb)

Table S2 (DOCX 14 kb)

## Data Availability

All data generated or analysed during this study are included in this published article [and its supplementary information files].
