# Supplementary material for: Polycyclic aromatic hydrocarbons in Mullus surmuletus from the Catania Gulf (Sicily, Italy): distribution and potential health risks
Source: Environ Sci Pollut Res Int. 2020 Oct 9;28(7):7756–65. doi: 10.1007/s11356-020-11052-z (PMC7854459; doi:10.1007/s11356-020-11052-z)
Supplement: Supplementary file 1 — (DOCX 14 kb) [file 11356_2020_11052_MOESM1_ESM.docx]

| Analyte | RT (min) | Target ion (m/z) | Qualifier ions (m/z) |
| --- | --- | --- | --- |
| Naphtalene-d8 (IS) | 8.85 | 136 | 68, 108 |
| Naphthalene | 8.89 | 128 | 51, 102 |
| Acenaphthylene | 14.60 | 152 | 76, 126 |
| Acenaphthene-d10 (IS) | 15.16 | 162 | 164, 80 |
| Acenaphthene | 15.27 | 153 | 76, 126 |
| Fluorene | 17.27 | 165 | 82, 139 |
| Phenanthrene-d10 (IS) | 20.87 | 188 | 94, 80 |
| Phenanthrene | 20.93 | 178 | 152, 89 |
| Anthracene | 21.14 | 178 | 152, 89 |
| Fluoranthene | 25.58 | 202 | 174, 101 |
| Pyrene | 26.39 | 202 | 174, 101 |
| Benzo(a)anthracene | 31.14 | 228 | 200, 114 |
| Chrysene-d12 (IS) | 31.18 | 240 | 236, 120 |
| Chrysene | 31.26 | 228 | 202, 114 |
| Benzo(b)fluoranthene | 35.05 | 252 | 126, 113 |
| Benzo(k)fluoranthene | 35.16 | 252 | 126, 113 |
| Benzo(a)pyrene | 36.11 | 252 | 126, 112 |
| Perylene-d12 (IS) | 36.33 | 264 | 260, 132 |
| Dibenzo(a,h)anthracene | 39.34 | 278 | 139, 125 |
| Indeno(1,2,3-cd)pyrene | 39.35 | 276 | 138, 125 |
| Benzo(g,h,i)perylene | 40.29 | 276 | 138, 124 |

**Table S1** Retention time, target and qualifier ions for PAHs. IS: Internal Standard
