# Supplementary material for: Polycyclic aromatic hydrocarbons in Mullus surmuletus from the Catania Gulf (Sicily, Italy): distribution and potential health risks
Source: Environ Sci Pollut Res Int. 2020 Oct 9;28(7):7756–65. doi: 10.1007/s11356-020-11052-z (PMC7854459; doi:10.1007/s11356-020-11052-z)
Supplement: Supplementary file 2 — (DOCX 14 kb) [file 11356_2020_11052_MOESM2_ESM.docx]

| Analyte | Internal standard used | Average recovery ± SD, %  (n = 5) |
| --- | --- | --- |
| Naphthalene | Naphtalene-d8 | 79 ± 3 |
| Acenaphthylene | Acenaphthene-d10 | 111 ± 7 |
| Acenaphthene | Acenaphthene-d10 | 96 ± 2 |
| Fluorene | Phenanthrene-d10 | 108 ± 3 |
| Phenanthrene | Phenanthrene-d10 | 116 ± 2 |
| Anthracene | Phenanthrene-d10 | 86 ± 4 |
| Fluoranthene | Chrysene-d12 | 102 ± 5 |
| Pyrene | Chrysene-d12 | 96 ± 2 |
| Benzo(a)anthracene | Chrysene-d12 | 102 ± 7 |
| Chrysene | Chrysene-d12 | 93 ± 4 |
| Benzo(b)fluoranthene | Perylene-d12 | 87 ± 3 |
| Benzo(k)fluoranthene | Perylene-d12 | 85 ± 4 |
| Benzo(a)pyrene | Perylene-d12 | 92 ± 2 |
| Dibenzo(a,h)anthracene | Perylene-d12 | 81 ± 2 |
| Indeno(1,2,3-cd)pyrene | Perylene-d12 | 75 ± 5 |
| Benzo(g,h,i)perylene | Perylene-d12 | 78 ± 6 |

**Table S2** Average recoveries (n = 5) and standard deviations (SD) for codfish samples spiked at 10 ng g^−1^
